# Supplementary material for: The interaction of InvF-RNAP is mediated by the chaperone SicA in Salmonella sp: an in silico prediction
Source: PeerJ. 2024 Mar 25;12:e17069. doi: 10.7717/peerj.17069 (PMC10977090; doi:10.7717/peerj.17069)
Supplement: Supplemental Information 1 [file peerj-12-17069-s001.pdf]

The Interaction of InvF-RNAP is mediated by the chaperone SicA in *Salmonella* sp: An *in silico* prediction

André Borges Farias[1], Daniel Cortés-Avalos[2], J. Antonio Ibarra[2], and Ernesto Pérez-Rueda[1]

[1]**Instituto de Investigaciones en Matemáticas Aplicadas y en Sistemas, Unidad Académica del Estado de Yucatán, Universidad Nacional Autónoma de México, Mérida, Yucatán, México**
[2]**Laboratorio de Genética Microbiana, Departamento de Microbiología, Escuela Nacional de Ciencias Biológicas, Instituto Politécnico Nacional, Ciudad de México, México**

Corresponding author:
André Borges Farias[1]

Email address: bfarias.andre@gmail.com

ABSTRACT

In this work we carried out an *in silico* analysis to understand the interaction between InvF-SicA and RNAP in the bacterium *Salmonella typhimurium* strain LT2. Structural analysis of InvF allowed the identification of three possible potential cavities for interaction with SicA. This interaction could occur with the TPR1 and TPR2 motifs in the two cavities located in the interface of the InvF and α-CTD of RNAP. Indeed, molecular dynamics simulations showed that SicA stabilizes the Helix-turn-Helix DNA-binding motifs, *i.e.* maintaining their proper conformation, mainly in the DNA Binding Domain (DBD). Finally, to evaluate the role of amino acids that contribute to protein-protein affinity, an alanine scanning mutagenesis approach, indicated that R177 and R181, located in the DBD motif, caused the greatest changes in binding affinity with α-CTD, suggesting a central role in the stabilization of the complex. However, it seems that the N-terminal region also plays a key role in the protein-protein interaction, especially the amino acid R40, since we observed conformational flexibility in this region allowing it to interact with interface residues. We consider that this analysis opens the possibility to validate experimentally the amino acids involved in protein-protein interactions and explore other regulatory complexes where chaperones are involved.

1 FIGURES

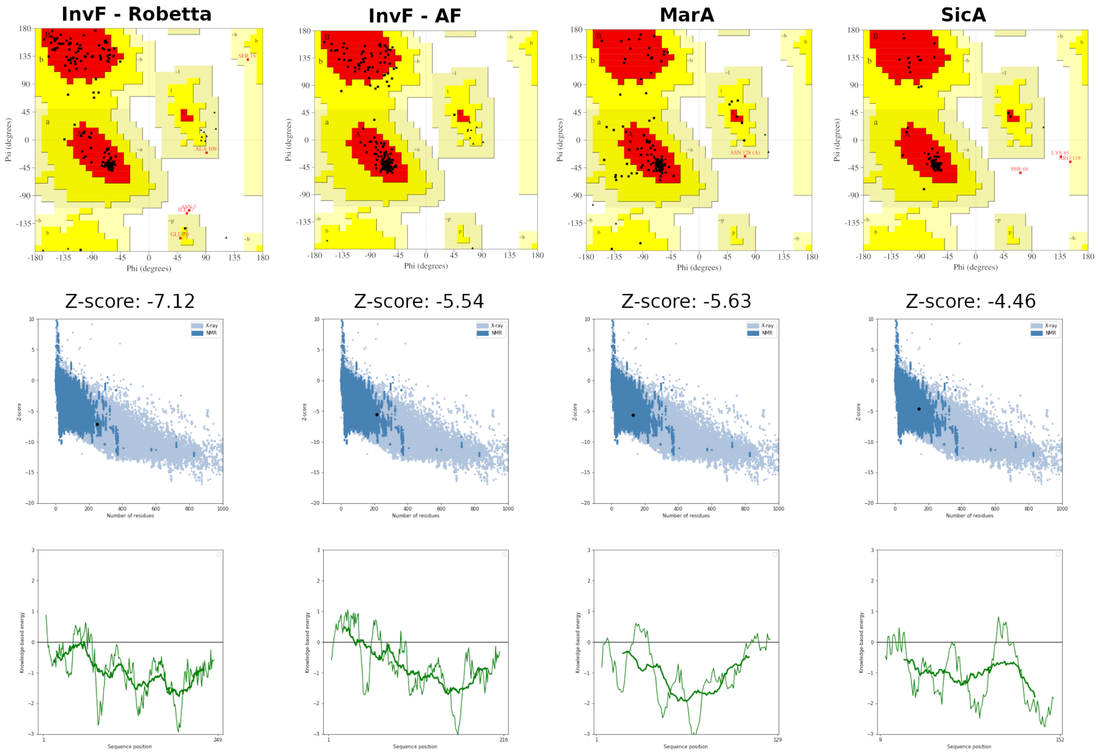

Figure S1. Ramachandran plot of InvF (**A**) and SicA (**B**). Local energy (**C**) and Z-score of InvF (**D**).

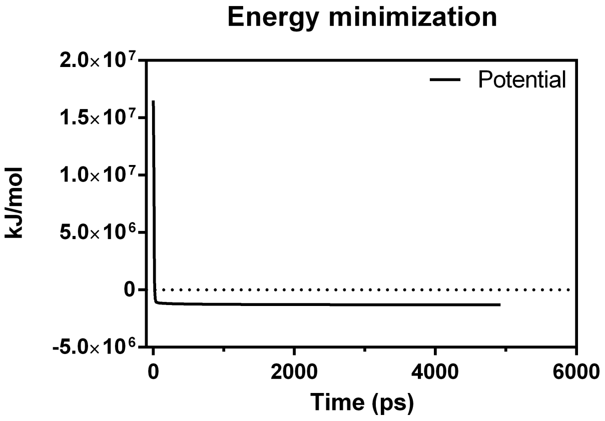

Figure S2. Energy minimization of InvF-α-CTD complex with steepest descent algorithm.

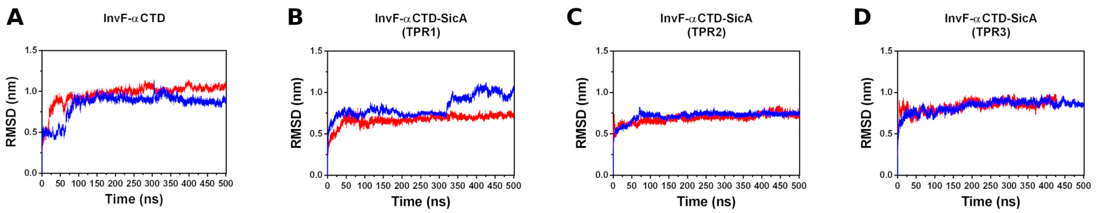

Figure S3. The root mean square deviation (RMSD) of residues from the backbone of InvF/α-CTD complex (**A**) and InvF/α-CTD/SicA complex interacting with TPR1 (**B**), TPR2 (**C**) and TPR3 (**D**), along molecular dynamics simulations. Simulations were performed in duplicates.

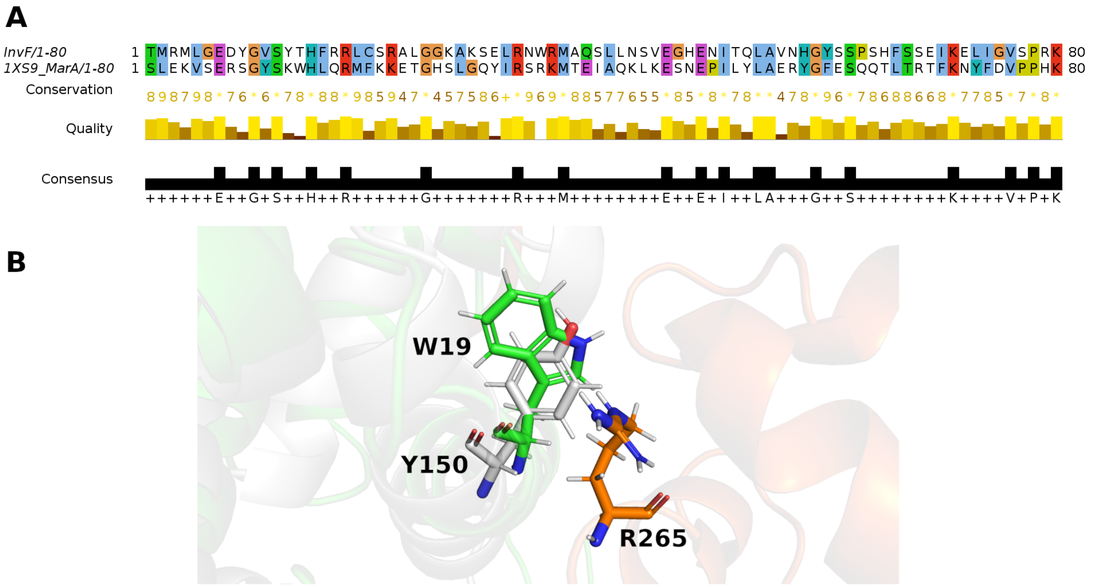

Figure S4. Sequence alignment of InvF and MarA shows 23.8 and 41.2 % of identity and similarity, respectively. (**A**). Cartoon representation of the structural alignment between InvF (grey) and MarA (green), highlighting the interaction between R265, from α-CTD, and conservative residue W19 (MarA) and Y150 (InvF) in an equivalent position (**B**).

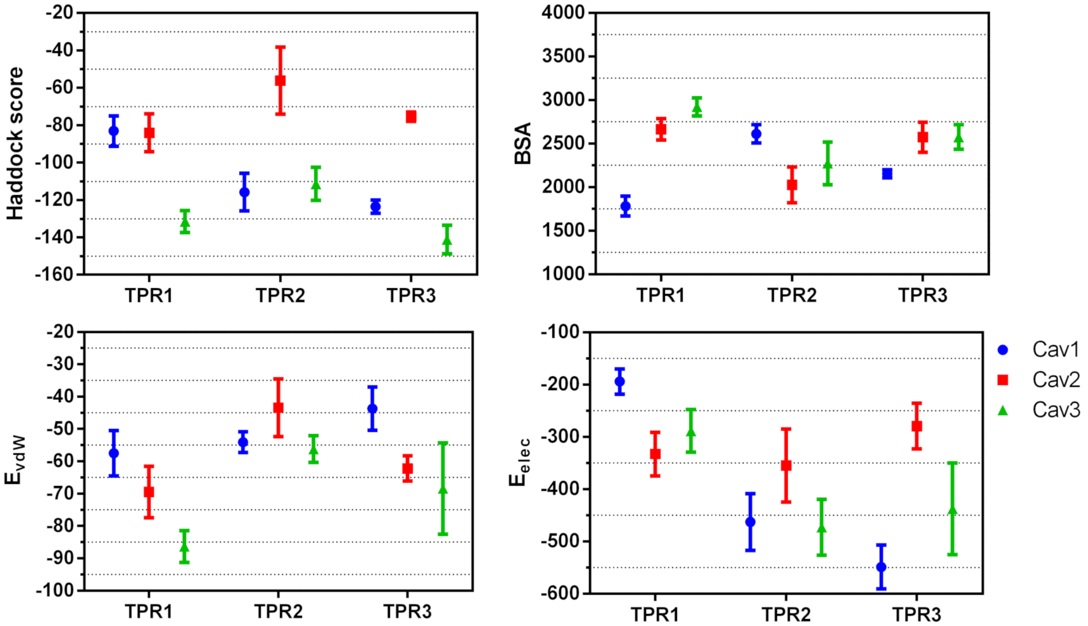

Figure S5. Haddock score values (upper left), the buried surface area (upper right), electrostatic (down left) and van der Waals (down right) contributions of the docking solutions.

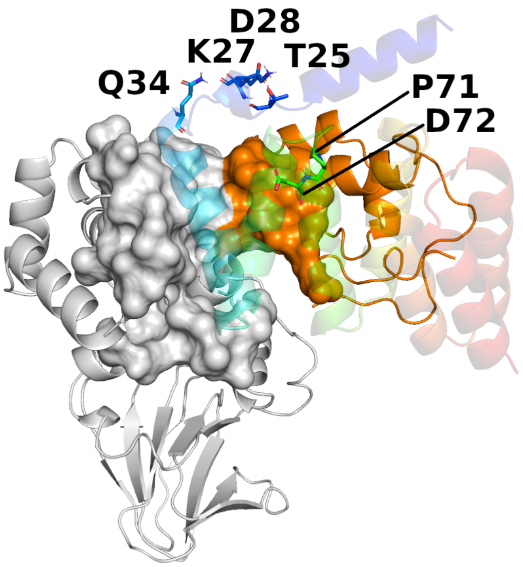

Figure S6. Cartoon representation of the protein complex InvF/α-CTD/SicA, highlighting residues described as relevant for interactions between c-di-GMP and SicA.

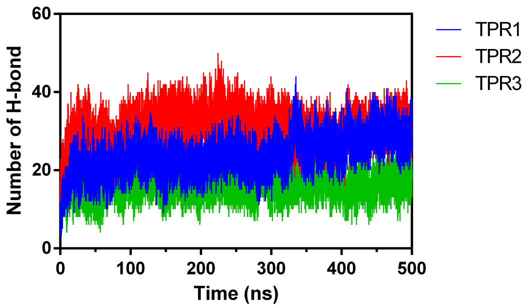

Figure S7. Amount of hydrogen bonds between InvF/α-CTD complex and SicA along molecular dynamics simulations.

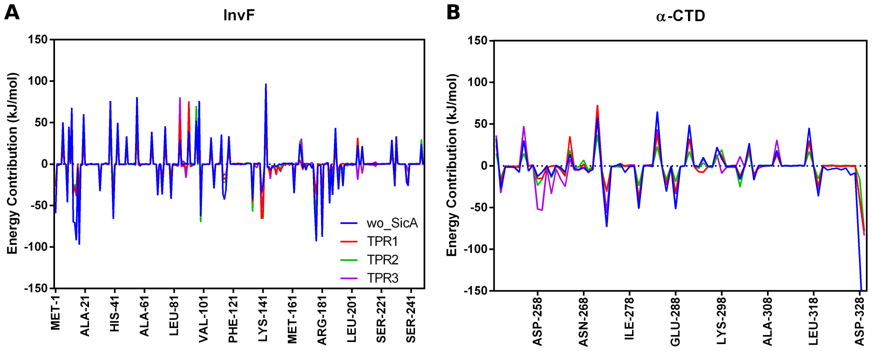

Figure S8. Contribution of energy per residue obtained by MM-PBSA of InvF (**A**) and α-CTD (**B**).

Table S1. Model validation by Ramachandran analysis.

	SicA		InvF Robetta		InvF AlphaFold		MarA 1xs9	
	N. res.	per. (%)	N. res.	per. (%)	N. res.	per. (%)	N. res.	per. (%)
Favoured regions	127	95.5	210	93.3	92.3	92.3	94	80.3
Allowed regions	3	2.3	10	4.4	15	7.7	22	18.8
Disallowed regions	2	1.5	2	0.9	0	0	0	0

2 TABLES

Table S2. Predicted cavities of InvF/RNAP complex by CavityPlus.

Cavity	Pred. Max. pKd	Pred. avg. pKd	Drugscore	Druggability
1	11.79	6.66	-33.00	Medium
2	11.17	6.45	4184.00	Strong
3	9.79	5.97	-163.00	Medium
4	9.21	5.77	-1165.00	Weak
5	8.97	5.69	-238.00	Weak
6	8.80	5.63	-1178.00	Weak
7	8.72	5.61	-203.00	Weak
8	8.59	5.56	-871.00	Weak
9	8.40	5.50	-904.00	Weak
10	7.95	5.35	606.00	Strong
11	7.31	5.12	-768.00	Weak
12	7.00	5.02	-1039.00	Weak
13	6.53	4.86	-348.00	Weak
14	5.43	4.48	-1429.00	Weak

Table S3. Docking results of SicA in Cavity 1. The standard deviation (SD) is represented between parentheses.

	Cluster	HADDOCK score	Cluster size	Van der Waals energy	Electrostatic energy	Desolvation energy	Restraints violation energy	BSA
TPR1	2	-83.1 (±8.1)	22	-57.5 (±7.0)	-194.1 (±24.0)	-4.5 (±3.8)	177.9 (±71.78)	1781.8 (±113.2)
	13	-78.2 (±10.8)	5	-41.2 (±8.5)	-266.2 (±39.5)	-9.7 (±2.7)	259.3 (±30.77)	1666.0 (±121.5)
	9	-77.4 (±7.9)	7	-63.2 (±4.7)	-164.1 (±41.4)	-9.8 (±2.0)	284.9 (±57.87)	2040.2 (±96.5)
	7	-77.4 (±3.8)	11	-57.4 (±7.4)	-178.5 (±28.0)	-8.6 (±2.3)	243.8 (±5.88)	1960.0 (±67.1)
	6	-73.4 (±4.4)	13	-51.7 (±5.5)	-191.8 (±29.2)	-3.7 (±2.6)	203.1 (±31.57)	1782.1 (±48.4)
	4	-68.2 (±4.9)	14	-43.2 (±6.8)	-188.2 (±45.3)	-6.0 (±2.6)	186.3 (±53.31)	1800.3 (±93.5)
	3	-67.9 (±2.8)	21	-46.4 (±3.7)	-116.3 (±9.8)	-14.7 (±2.2)	165.0 (±29.09)	1649.0 (±44.9)
	1	-66.3 (±3.2)	41	-48.2 (±4.5)	-149.9 (±10.5)	-9.2 (±1.4)	211.4 (±40.58)	1538.7 (±48.8)
	5	-64.0 (±8.4)	14	-33.4 (±7.6)	-245.0 (±70.6)	-10.0 (±3.5)	283.5 (±41.80)	1618.7 (±76.0)
	8	-62.1 (±7.2)	8	-39.2 (±4.8)	-192.1 (±43.6)	-6.7 (±3.0)	221.4 (±40.78)	1396.1 (±56.0)
TPR2	9	-115.8 (±10.1)	5	-54.1 (±3.2)	-462.9 (±54.4)	7.5 (±2.5)	234.2 (±61.21)	2610.6 (±105.2)
	1	-96.9 (±7.8)	57	-34.0 (±6.8)	-428.7 (±29.2)	1.2 (±2.0)	216.8 (±39.62)	1896.2 (±77.3)
	5	-96.6 (±14.1)	10	-46.2 (±4.2)	-409.4 (±69.1)	8.1 (±2.8)	233.5 (±74.39)	2196.1 (±256.0)
	11	-95.9 (±6.4)	4	-64.2 (±8.5)	-308.0 (±22.2)	-1.0 (±0.8)	308.2 (±85.20)	2402.0 (±195.5)
	10	-88.5 (±20.8)	4	-37.8 (±1.1)	-363.3 (±139.0)	-2.8 (±6.1)	247.6 (±59.09)	2009.4 (±297.0)
	2	-86.4 (±9.0)	30	-38.7 (±7.0)	-402.8 (±10.9)	10.3 (±4.3)	225.9 (±56.83)	1650.6 (±193.8)
	4	-83.7 (±5.4)	13	-37.6 (±9.9)	-362.8 (±65.8)	7.6 (±4.7)	188.5 (±29.35)	1921.6 (±217.2)
	8	-81.5 (±4.0)	5	-48.1 (±7.9)	-277.7 (±42.8)	4.1 (±4.0)	180.4 (±32.36)	1978.0 (±101.3)
	3	-73.8 (±9.6)	13	-43.5 (±6.6)	-282.2 (±98.6)	-0.0 (±3.4)	261.5 (±44.31)	1921.2 (±20.7)
	6	-65.0 (±4.2)	7	-57.8 (±3.6)	-154.6 (±37.5)	-11.7 (±4.3)	354.1 (±45.18)	2221.1 (±107.7)
TPR3	4	-123.5 (±3.5)	10	-43.7 (±6.7)	-548.8 (±41.9)	11.0 (±2.5)	189.7 (±12.98)	2152.8 (±46.3)
	3	-90.4 (±11.7)	12	-55.6 (±9.6)	-236.1 (±30.1)	-7.7 (±1.2)	200.8 (±64.42)	2286.6 (±230.8)
	2	-82.7 (±3.5)	14	-35.8 (±4.9)	-377.2 (±32.9)	5.0 (±3.3)	235.5 (±12.90)	2033.7 (±171.7)
	5	-76.2 (±1.9)	9	-49.8 (±2.4)	-185.4 (±14.4)	-11.7 (±2.4)	223.8 (±26.15)	2090.6 (±91.5)
	13	-70.3 (±10.3)	4	-55.5 (±9.0)	-136.3 (±17.5)	-18.8 (±4.3)	313.5 (±39.67)	2222.5 (±136.3)
	8	-69.1 (±4.0)	6	-51.6 (±3.4)	-171.8 (±17.0)	0.7 (±1.8)	160.9 (±23.91)	1799.0 (±106.7)
	1	-69.0 (±5.6)	83	-33.1 (±3.2)	-255.7 (±40.1)	0.0 (±1.7)	152.2 (±54.35)	1490.5 (±111.0)
	11	-63.1 (±14.6)	4	-45.5 (±6.5)	-177.1 (±68.4)	-8.3 (±5.1)	261.0 (±37.86)	1981.9 (±152.3)
	7	-61.0 (±0.8)	6	-43.9 (±3.7)	-181.8 (±36.1)	2.3 (±2.2)	170.7 (±62.87)	1546.6 (±65.5)
	10	-58.3 (±11.5)	5	-42.4 (±4.8)	-121.5 (±55.0)	-9.4 (±4.9)	177.4 (±46.45)	1754.6 (±118.4)

Table S4. Docking results of SicA in Cavity 2. The standard deviation (SD) is represented between parentheses.

	Cluster	HADDOCK score	Cluster size	Van der Waals energy	Electrostatic energy	Desolvation energy	Restraints violation energy	BSA
TPR1	7	-84.1 (± 10.1)	7	-69.5 (±7.9)	-332.9 (±41.6)	1.3 (±3.8)	507.2 (±77.56)	2662.7 (±122.4)
	4	-69.4 (±5.5)	13	-61.9 (±2.9)	-231.3 (±47.3)	-11.1 (±5.5)	498.0 (±36.46)	2273.3 (±148.2)
	12	-67.1 (±6.4)	5	-70.2 (±5.9)	-230.5 (±25.5)	-3.0 (±4.1)	522.0 (±19.50)	2511.8 (±79.9)
	8	-50.4 (±16.2)	6	-51.6 (±6.5)	-284.5 (±40.0)	-2.2 (±2.0)	602.2 (±112.83)	1794.9 (±50.9)
	2	-48.0 (±4.1)	21	-52.2 (±1.2)	-271.4 (±15.2)	0.0 (±1.0)	585.0 (±37.82)	1999.5 (±111.1)
	9	-43.1 (±6.0)	6	-38.6 (±6.2)	-379.2 (±30.1)	9.5 (±0.9)	618.2 (±52.44)	1653.5 (±59.5)
	1	-40.6 (±2.7)	37	-58.0 (±5.0)	-214.7 (±41.9)	1.4 (±2.1)	590.0 (±43.00)	2036.5 (±112.9)
	11	-39.7 (±4.0)	5	-46.2 (±5.0)	-256.6 (±24.5)	-10.5 (±2.4)	683.6 (±105.93)	2160.6 (±294.8)
	6	-39.4 (±5.8)	8	-37.0 (±3.1)	-384.6 (±8.1)	10.2 (±2.8)	643.9 (±47.01)	1546.7 (±58.8)
	14	-31.7 (±10.7)	4	-22.1 (±12.5)	-333.2 (±29.2)	5.3 (±0.9)	518.1 (±70.55)	1569.5 (±74.9)
TPR2	5	-56.2 (± 17.9)	7	-43.4 (± 8.9)	-354.7 (± 70.0)	-2.2 (± 1.6)	603.7 (± 30.52)	2026.5 (± 205.6)
	6	-51.6 (± 8.4)	5	-71.5 (± 6.5)	-226.6 (± 79.5)	-5.2 (± 1.9)	704.5 (± 25.35)	2455.3 (± 81.6)
	4	-41.8 (± 15.8)	9	-53.2 (± 4.5)	-231.8 (± 25.1)	0.3 (± 4.0)	574.6 (± 111.35)	2197.6 (± 201.8)
	1	-34.5 (± 4.2)	20	-52.1 (± 4.7)	-251.0 (± 56.5)	4.9 (± 2.9)	628.7 (± 86.34)	2074.8 (± 38.0)
	10	-14.0 (± 8.2)	4	-31.6 (± 7.5)	-216.3 (± 50.2)	-4.2 (± 3.1)	650.9 (± 55.13)	1615.8 (± 100.0)
	2	-12.3 (± 6.6)	13	-47.7 (± 4.2)	-149.6 (± 53.4)	4.9 (± 3.1)	604.6 (± 33.91)	1802.6 (± 68.1)
	9	-10.4 (± 11.1)	4	-43.5 (± 4.7)	-167.1 (± 44.3)	6.8 (± 5.1)	596.6 (± 29.22)	1452.2 (± 101.1)
	3	-2.6 (± 1.4)	13	-52.8 (± 2.4)	-148.8 (± 20.4)	8.2 (± 2.1)	717.6 (± 29.31)	1686.0 (± 57.5)
	8	1.6 (± 7.1)	4	-49.1 (± 2.6)	-110.2 (± 19.8)	1.9 (± 1.5)	709.3 (± 54.32)	1389.8 (± 79.4)
	7	6.4 (± 9.4)	4	-46.5 (± 7.1)	-140.7 (± 18.2)	2.3 (± 2.8)	787.4 (± 49.67)	1974.4 (± 110.1)
TPR3	1	-75.4 (± 2.5)	32	-62.2 (± 3.9)	-279.2 (± 43.4)	-3.7 (± 3.1)	462.4 (± 64.14)	2572.9 (± 173.4)
	7	-47.9 (± 11.6)	7	-59.8 (± 8.6)	-138.6 (± 9.9)	-12.1 (± 2.8)	517.5 (± 123.63)	2124.7 (± 158.0)
	2	-38.5 (± 3.7)	20	-41.5 (± 6.8)	-312.8 (± 26.6)	7.5 (± 2.1)	581.0 (± 22.23)	1798.7 (± 38.1)
	11	-33.3 (± 17.0)	4	-52.3 (± 8.5)	-165.3 (± 27.4)	-4.6 (± 3.1)	566.7 (± 68.56)	1837.3 (± 183.5)
	5	-32.5 (± 2.3)	10	-46.2 (± 7.1)	-176.8 (± 42.4)	-3.1 (± 3.4)	522.3 (± 44.72)	1770.1 (± 137.6)
	10	-28.0 (± 18.3)	4	-49.4 (± 8.2)	-121.4 (± 36.3)	-3.2 (± 5.6)	489.3 (± 32.74)	1807.8 (± 148.4)
	13	-27.6 (± 15.6)	4	-39.5 (± 5.4)	-195.3 (± 67.0)	6.4 (± 3.5)	445.6 (± 34.05)	1677.9 (± 155.8)
	4	-26.8 (± 4.9)	10	-43.8 (± 6.6)	-249.1 (± 73.2)	3.9 (± 5.2)	629.5 (± 32.16)	1881.1 (± 71.7)
	3	-26.4 (± 3.3)	12	-38.7 (± 4.5)	-183.9 (± 27.7)	5.2 (± 3.3)	439.3 (± 60.96)	1520.3 (± 62.5)
	9	-23.5 (± 5.3)	5	-39.9 (± 2.7)	-167.5 (± 50.1)	-0.6 (± 1.4)	505.0 (± 111.05)	1595.4 (± 38.7)

Table S5. Docking results of SicA in Cavity 3. The standard deviation (SD) is represented between parentheses.

	Cluster	HADDOCK score	Cluster size	Van der Waals energy	Electrostatic energy	Desolvation energy	Restraints violation energy	BSA
TPR1	3	-131.5 (± 5.8)	26	-86.3 (± 4.9)	-288.6 (± 40.8)	-3.4 (± 2.3)	159.3 (± 33.58)	2923.4 (± 103.7)
	9	-96.7 (± 3.8)	6	-56.4 (± 2.5)	-252.3 (± 14.5)	-12.1 (± 1.5)	221.6 (± 42.38)	2046.3 (± 69.1)
	2	-91.3 (± 6.1)	29	-63.6 (± 2.7)	-179.7 (± 19.7)	-5.4 (± 2.2)	136.6 (± 18.77)	2010.1 (± 75.0)
	5	-86.5 (± 12.0)	10	-60.1 (± 5.8)	-178.2 (± 28.9)	-8.4 (± 2.9)	176.8 (± 46.66)	2118.1 (± 181.9)
	1	-85.8 (± 1.2)	42	-67.0 (± 11.0)	-156.0 (± 37.1)	-11.0 (± 1.7)	233.5 (± 59.84)	2088.2 (± 124.3)
	8	-85.4 (± 8.9)	8	-63.5 (± 2.6)	-193.8 (± 35.9)	-4.0 (± 1.5)	207.7 (± 48.48)	1895.4 (± 96.8)
	4	-76.3 (± 6.4)	25	-64.9 (± 7.3)	-136.3 (± 42.3)	-3.7 (± 3.0)	196.4 (± 30.07)	2024.7 (± 132.3)
	6	-65.8 (± 8.8)	8	-58.1 (± 9.1)	-112.3 (± 19.5)	-4.4 (± 4.0)	192.3 (± 35.74)	2126.3 (± 152.1)
	10	-64.0 (± 7.4)	5	-65.2 (± 4.8)	-108.6 (± 2.5)	-7.2 (± 5.0)	301.0 (± 47.10)	1954.8 (± 79.2)
	11	-58.1 (± 3.3)	5	-45.4 (± 1.6)	-221.6 (± 20.4)	5.9 (± 1.4)	256.9 (± 57.41)	1872.1 (± 89.8)
TPR2	5	-111.4 (± 8.8)	10	-56.2 (± 4.1)	-472.9 (± 53.4)	10.8 (± 2.0)	285.9 (± 23.18)	2273.7 (± 246.2)
	2	-106.8 (± 4.8)	32	-58.7 (± 14.4)	-329.5 (± 85.6)	3.9 (± 4.4)	139.5 (± 46.97)	2369.4 (± 103.7)
	1	-104.2 (± 2.5)	56	-48.1 (± 5.2)	-437.1 (± 28.9)	5.5 (± 1.9)	258.4 (± 50.37)	2096.8 (± 72.1)
	9	-93.9 (± 12.8)	5	-49.4 (± 8.5)	-398.6 (± 30.1)	7.2 (± 4.6)	279.9 (± 29.57)	2372.7 (± 259.0)
	12	-93.1 (± 9.9)	4	-54.6 (± 10.1)	-349.9 (± 54.9)	2.6 (± 4.4)	288.6 (± 47.99)	2347.3 (± 263.1)
	4	-88.9 (± 1.9)	12	-58.8 (± 6.1)	-270.7 (± 32.4)	0.6 (± 2.4)	235.1 (± 69.83)	2393.4 (± 245.7)
	8	-85.4 (± 3.5)	5	-40.7 (± 3.4)	-388.5 (± 36.8)	13.0 (± 3.2)	199.8 (± 26.52)	2239.4 (± 98.6)
	11	-82.9 (± 14.8)	4	-46.9 (± 2.1)	-284.9 (± 73.2)	5.5 (± 4.0)	154.8 (± 29.94)	2102.2 (± 104.2)
	6	-80.2 (± 9.2)	9	-56.4 (± 3.2)	-311.2 (± 45.5)	0.5 (± 2.0)	378.9 (± 67.07)	2352.2 (± 96.8)
	7	-73.8 (± 11.0)	7	-60.2 (± 3.6)	-285.4 (± 49.2)	2.6 (± 1.3)	409.0 (± 50.73)	2298.0 (± 196.7)
TPR3	1	-141.2 (± 7.6)	49	-68.4 (± 14.1)	-437.7 (± 87.7)	3.7 (± 5.9)	109.7 (± 55.70)	2574.8 (± 141.3)
	2	-129.2 (± 3.1)	27	-68.3 (± 4.9)	-378.0 (± 32.9)	3.5 (± 4.4)	112.6 (± 52.03)	2471.2 (± 143.4)
	5	-99.2 (± 6.1)	11	-54.3 (± 9.1)	-347.7 (± 47.2)	9.5 (± 7.4)	151.5 (± 55.70)	2226.0 (± 191.1)
	7	-98.6 (± 2.8)	10	-50.6 (± 4.0)	-310.5 (± 44.5)	-2.3 (± 4.0)	162.9 (± 18.85)	2172.1 (± 84.5)
	9	-94.1 (± 20.1)	7	-63.0 (± 13.6)	-230.4 (± 29.3)	-2.6 (± 8.8)	175.6 (± 43.04)	2753.7 (± 357.7)
	4	-92.2 (± 5.9)	24	-49.8 (± 9.2)	-326.9 (± 40.4)	6.8 (± 2.8)	162.5 (± 70.83)	1954.9 (± 141.9)
	3	-91.4 (± 4.2)	26	-60.9 (± 4.5)	-306.9 (± 45.0)	3.6 (± 1.6)	272.6 (± 23.95)	2167.2 (± 61.4)
	6	-88.5 (± 6.3)	10	-52.3 (± 3.1)	-288.4 (± 18.0)	4.5 (± 2.7)	169.7 (± 55.48)	2163.5 (± 75.2)
	11	-86.7 (± 9.9)	4	-39.2 (± 10.3)	-387.5 (± 42.4)	12.9 (± 3.8)	170.8 (± 65.09)	2057.6 (± 145.1)
	8	-80.5 (± 12.0)	9	-42.6 (± 3.7)	-346.7 (± 56.7)	8.8 (± 5.0)	225.8 (± 25.04)	2010.3 (± 79.6)
